# Allee effects limit coral fertilization success

**DOI:** 10.1073/pnas.2418314121

**Published:** 2024-12-16

**Authors:** Peter J. Mumby, Greta Sartori, Elizabeth Buccheri, Cinzia Alessi, Hannah Allan, Christopher Doropoulos, Geraldine Rengiil, Gerard Ricardo

**Affiliations:** ^a^Marine Spatial Ecology Lab, School of the Environment, The University of Queensland, St. Lucia, QLD 4072, Australia; ^b^Palau International Coral Reef Center, Koror 96940, Republic of Palau; ^c^Environment Department, Commonwealth Scientific, Industrial, and Research Organisation, St. Lucia, QLD 4067, Australia

**Keywords:** reproduction, coral reef, threshold, resilience, spawning

## Abstract

Coral populations are being progressively thinned by climate change, which elevates the risk of reproductive failure from Allee effects during fertilization. Studies have shown that fertilization success improves during more intense and synchronized spawning, but the local dependence of fertilization on coral density remains unknown in wild populations. Here, we measure the fertilization success of individual colonies of the common table coral, *Acropora hyacinthus* in Palau, Micronesia. We find strong evidence of spatial Allee effects such that fertilization averaged 30% when colonies were in close proximity (<0.5 m), but this declined rapidly to less than 10% at a separation of 10 m and virtually zero by 15 to 20 m. The distance of a nearest neighbor conspecific, gravid colony is a better predictor of observing Allee effects than local colony density measured at several alternative scales (2 to 10 m). Spawning synchrony increases when corals are in close proximity, which may reinforce Allee effects as reproductive colonies become further separated. Fertilization success declined nearly threefold on a second night of spawning with higher wind despite there being more intensive spawning of colonies, highlighting the potential importance of prevailing weather in driving the outcome of mass spawning events. Overall, we find that the local population of *A. hyacinthus* achieved 71% of its fertilization potential. Further studies of spatial patchiness in coral populations are needed to generalize the likelihood of Allee effects, how climate change impacts reproductive function on reefs, and opportunities for restoration to safeguard reproductive success by reconnecting depleted coral populations.

## Significance

Climate change is reducing population densities of corals, which risks the onset of reproductive collapse once colonies become too far apart to achieve successful fertilization. Working with a natural population of a widespread, reef-building coral, we find that critical thresholds of colony spacing lie between 10 and 15 m. Our results provide a framework to monitor the risks of Allee effects in coral populations and offer a target for restoration activities such that reproduction is protected.

The impact of climate change on biodiversity loss is considered to be greater in marine ecosystems than their terrestrial or freshwater counterparts (1). The disruption caused by rising sea temperatures and acute heatwaves affects virtually every marine biome on Earth (2). While climate change acts through myriad mechanisms, those that elicit nonlinear population responses and tipping points are exceptionally dangerous because they can manifest suddenly and be difficult to reverse (3). Like many benthic invertebrates, tropical hard corals are susceptible to both acute heatwaves and nonlinear dynamics (4). Moreover, as sessile animals, corals are theoretically vulnerable to component Allee effects on reproduction that arise when mates are too far apart to achieve successful fertilization (5). If true, then heatwave-driven coral bleaching events, that reduce population density (6), could create further nonlinearities and even negative population growth.

Most corals are hermaphroditic broadcast spawners (7), releasing bundles of eggs and sperm during periodic mass spawning events (8). Successful fertilization of eggs requires a sufficiently high concentration of sperm (9–12). Yet, because broadcast spawning corals are sessile organisms, sperm concentration is likely influenced by the distance among colonies, total gamete output (9), and the synchrony of gamete release (13, 14). Such so-called Allee effects (15), which in this case refer to the importance of the density and proximity among reproductive colonies, can limit reproductive success, recruitment, and therefore population recovery (16, 17). Allee effects pose a significant threat to the future resilience of coral reefs because the frequency and intensity of heatwaves are increasing (18) and will likely increase the average distance among surviving colonies.

Evidence for the dependency of fertilization success on the proximity among mobile invertebrates has yielded a diversity of results with high fertilization occurring at scales of a few centimeters to tens of meters (19–21). The importance of colony spacing in sessile organisms, like corals, is less clear, with few empirical studies being conducted. For instance, the majority of fertilization of the gonochoric gorgonians, *Plexaura kuna* and *Pseudoplexaura porosa*, appears to occur within 1 m of the female colony, if it occurs at all (13, 22). For Scleractinian corals, which are dominated by hermaphroditic spawners on Indo-Pacific coral reefs (7), studies on wild populations are limited. Observations of *Montipora digitata* fertilization from water samples in the field indicate that fertilization success can decrease by orders of magnitude during minor spawning nights (i.e., when 3 to 16% of the population spawn) compared to the major spawning night (i.e., 71% of population spawning; 9). Similarly, the average proportion of fertilized eggs in *Orbicella* spp. was found to be greater on nights where more corals were observed spawning (14), implying the existence of Allee effects. Yet, how colony density and spacing influences such patterns requires mechanistic understanding with a view to defining minimum colony densities required to alleviate Allee effects and sustain coral reef reproductive function.

Here, we quantify the colony-level fertilization success of the reef-building table coral *Acropora hyacinthus* during a natural spawning event in Palau, Micronesia. *A. hyacinthus* is found in a diversity of physical environments and its fast growth and large size allow it to contribute strongly to many ecosystem functions (23) and drive rapid reef recovery in Palau (24) and elsewhere (25, 26). Our primary hypothesis is that fertilization success declines with increasing isolation of colonies owing to limitations on sperm-egg cross-fertilization between neighboring colonies. We also explored two other factors that impact fertilization success: a) the intensity of gamete release, which often differs between successive spawning nights (9), and b) the synchrony of gamete release, which is motivated by a Caribbean study that observed higher synchronization in corals in close proximity (14). We explored whether this is also the case in our common Pacific coral species. Finally, we considered how our expected measurement of Allee effects could be expressed as a reef-level metric to facilitate future comparisons.

## Results

**Fig. 1.**
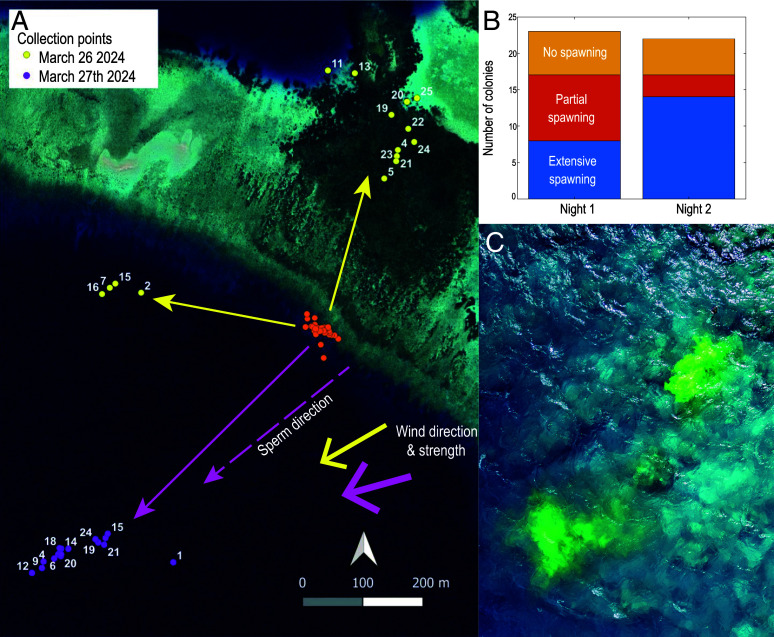
Spawning behavior of *A. hyacinthus* on the 26th (night 1) and 27th (night 2) of March 2024 showing (*A*) collection points of floating egg containers after ~1 h of drifting, (*B*) trends in spawning intensity and (*C*) use of fluorescein dye to track sperm direction at equivalent tide and wind to night 2. Orange circles mark the release point of tagged colonies. Sperm direction also shown in (*A*).

Spawning occurred on two successive nights, the 26th and 27th of March 2024. In each case, spawning peaked at approximately 21:00 local time, which was 35 min and 5 min after high tide on each night, respectively (with tidal heights 5.8 m and 5.7 m). The prevalence of spawning was moderately high on the first night with 74% of colonies releasing bundles but only 35% exhibiting extensive spawning across each colony (Fig. 1*B*). A similar proportion of colonies released on the second night (77%), but nearly twice as many underwent extensive spawning (64%), such that overall spawning intensity was greater (marginally significant at *P* = 0.056).

To quantify colony-level fertilization success, we placed an egg container just above each of 26 colonies. Containers were released once they had gathered at least 1 cm^3^ of egg bundles and floated upward to drift just under the sea surface. The containers retained eggs but were permeable to sperm. The first night typified spawning conditions with light winds stemming from the northeast (59 to 66°) with an average speed of 1.9 ms^−1^ and gusts up to 4.3 ms^−1^ between 20:00 and 23:00. However, wind increased on the second, more intensive, night of spawning, at 2.3 ms^−1^ with gusts exceeding 5.0 ms^−1^, stemming from the northeast (70 to 73°). The trajectories of egg containers differed between nights (Fig. 1*A*). On the first night, the eggs traveled in two directions—a larger group of samples passed across the reef flat and a smaller group tracked just off the edge of the forereef. On the second night, egg containers mostly formed a large group that resembled a frontal slick and moved offshore. Average velocities were 0.1 (±0.02 SD) ms^−1^ on night 1 and 0.15 (±0.01 SD) ms^−1^ on night 2. On the 29th of March we replicated the tidal and wind conditions of night 2 and undertook two releases of fluorescein dye to simulate sperm dispersal (Fig. 1*C*). These tracked virtually the same trajectory as the containers (235° for containers vs. 240°) and moved at comparable speed (0.13 ms^−1^ SD 0.05).

### Neighborhood Metrics, Spawning Intensity, and Synchrony on Fertilization Rate.

The most parsimonious model of fertilization success included the simplest measure of intercolony distance—nearest neighbor—which extended to a distance of 19 m (Table 1). Confining the proximity metric to just the number of colonies within a 2 m radius remained significant, but the model fit was considerably poorer (Table 1). Extending the density metric to consider colonies within either 5 m or 10 m led to nonsignificant effects. A weighted metric intended to represent the local area of spawning colonies, weighted by distance within a 10 m radius, yielded significant explanatory power but a poorer fit than a simple nearest neighbor (AIC 249.5 vs. 241.3, Table 1).

**Table 1.** Candidate models of colony-level fertilization success for *A. hyacinthus* considering several intercolony proximity metrics, night of spawning, and spawning synchrony

Proximity metric	Night	Other fixed	Coefficients (SE)	*P*	AIC
Nearest neighbor (nn)	Yes (n1, n2)	none	nn = −0.16 (0.05) n2 = −1.01 (0.30)	<0.001 <0.001	241.3
Nearest neighbor	Yes	Release time (synchrony)	nn = −0.20 (0.07) nn = −2.05 (0.71) n1:time = 3.00 (1.31) n2:time = 6.61 (5.27) n1:time^2^ = 1.17 (1.23) n2:time^2^ = −4.25 (3.85)	<0.01 <0.01 0.02 ns (0.21) ns (0.34) ns (0.27)	243.3
Density (D) within 2 m	Yes	none	D = 0.21 (0.10) n2 = −1.01 (0.32)	0.04 <0.01	248.7
Weighted colony area (A) within 10 m	Yes	none	A = 0.03 (0.02) n2 = −0.98 (0.33)	m. 0.07 <0.01	249.5
Density (D) within 5 m	Yes	none	D = 0.04 (0.03) n2 = −1.05 (0.33)	ns (0.23) <0.01	250.9
Density (D) within 10 m	Yes	none	D = 0.005 (0.01) n2 = −1.05 (0.33)	ns (0.66) <0.01	252.1

“m.” is used to indicate marginal significance, whereas “n.s.” indicates nonsignificant. The intercept for the best-fitting model is −0.849 with (0.22 SE). All coefficients are untransformed.

**Fig. 2.**
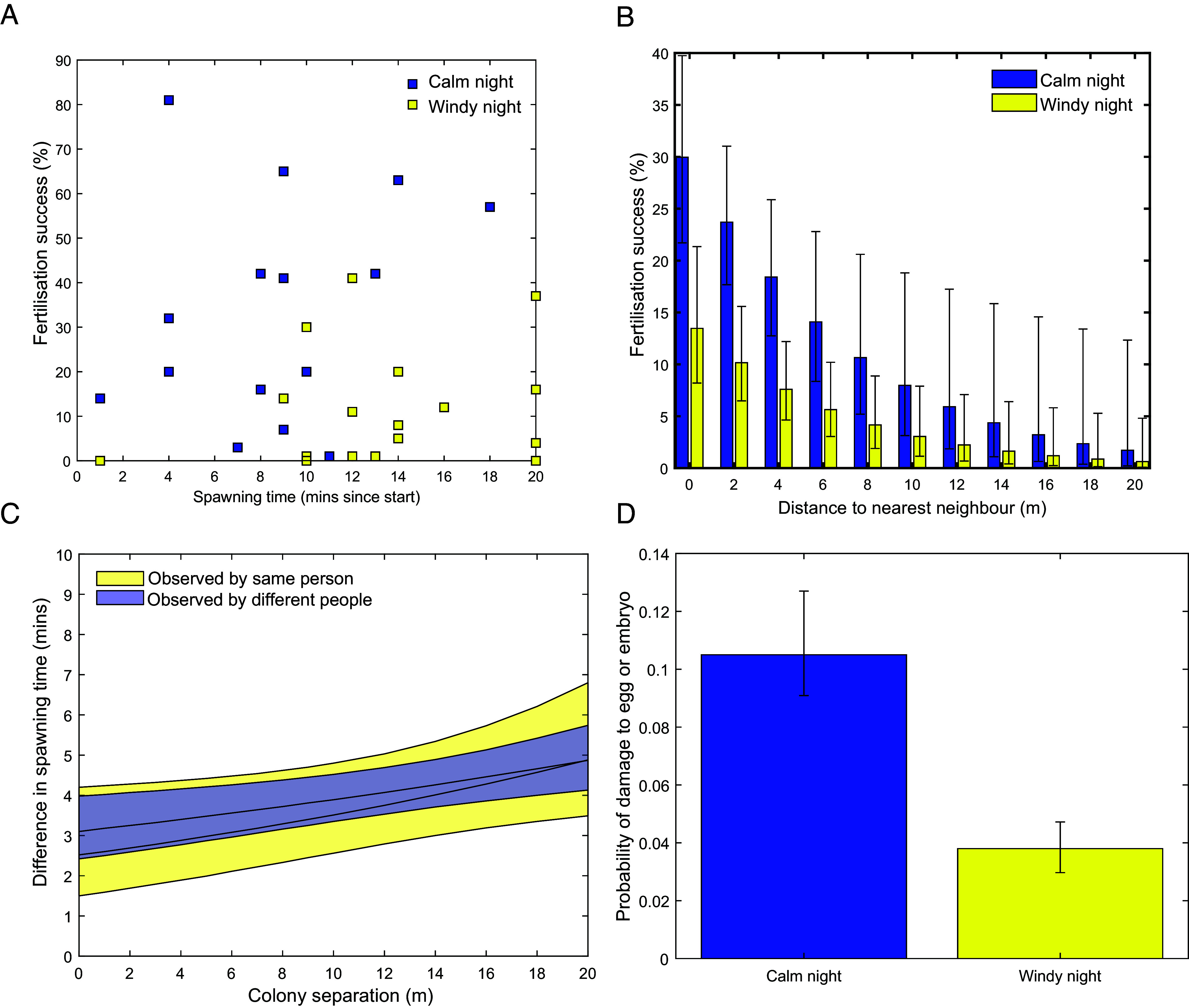
Key results of study showing the relationships between spawning time and fertilization success on successive nights (*A*), Allee effects of distance between colonies on fertilization success (*B*), the effects of colony distance on spawning synchrony (*C*), and the probability of encountering a damaged gamete in the egg container (*D*). All CI are 95%. Impacts on colony-level fertilization success on nights 1 (calm) and 2 (windy) revealing the weak positive impact of spawning synchrony on night 1 (*A*) and strong negative effects of the windy night and distance to the nearest neighboring colony (*B*). Error bars denote 95% CL.

Inclusion of the synchrony of spawning among colonies each evening failed to improve model fit (Table 1). The quadratic terms were both rejected though there was some evidence of an increase in fertilization success in later-spawning colonies on the first night (Fig. 2*A* and Table 1). Similarly, depth class did not influence fertilization success (*P* = 0.45). The random effect of colony identity (1-26) was close to zero (typically <10^−7^) and only retained for model completeness. Removal did not alter any fixed effects.

We also tested a posteriori whether the pattern of egg dispersal could explain fertilization success. To do so, we replaced the term “night” in the parsimonious model with the three primary outcomes of dispersal (Fig. 1). However, unlike night, dispersal was nonsignificant (*P* = 0.56).

Fertilization success declined rapidly with increasing distance between neighboring corals (Fig. 2*B*). At a close proximity of <1 m, modeled mean fertilization success was 30% during the first night but dropped to 10% within 8 to 10 m and 1% by 20 m. There was no evidence that the shape of this relationship changed from one night to the next because an interaction term between nearest neighbor and night was highly nonsignificant (*P* = 0.98). However, fertilization success was much lower on the second night with mean fertilization reaching only 40% that of the first night (mean 34% on night 1, 12% on night 2; Fig. 2*B*).

Spawning synchrony was greater among colonies that were located closer together (Fig. 2*C*). The fitted model predicted that synchrony declined by approximately 3 min over a distance of 20 m. Measurements of spawning synchrony were unaffected by whether the same or different observers recorded data (*P* = 0.14; see *SI Appendix*).

Given that the second night exhibited higher wind and lower fertilization success, we tested whether the expected increase in turbulence led to a higher percentage of damaged eggs and/or embryos, where damage included deformities and fragmentation. Yet we observed the opposite, with the probability of encountering a damaged gamete being nearly threefold higher on the first night (*P* < 0.001; Fig. 2*D*).

**Fig. 3.**
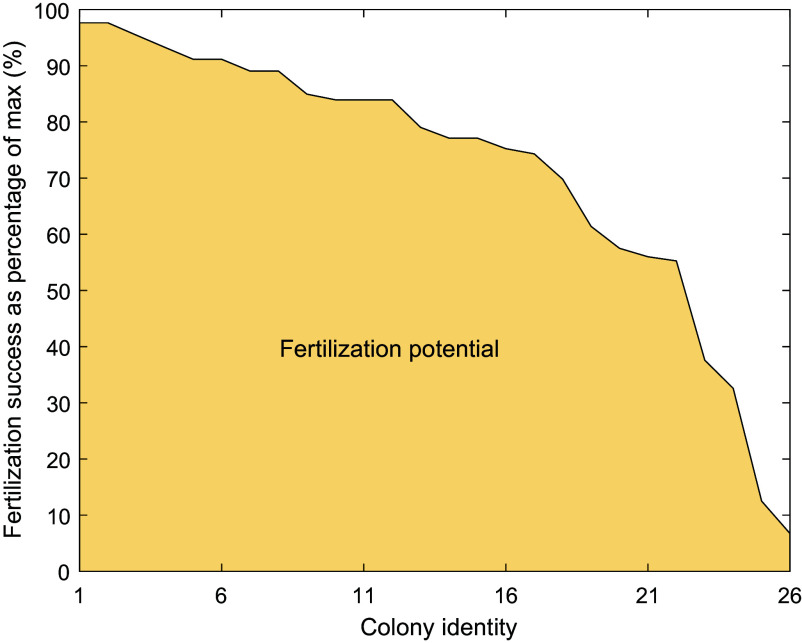
Representation of the metric for the risk of Allee effects on *A. hyacinthus* at our study reef. Colonies are ordered from that with the closest nearest neighbor to the most isolated. The *y*-axis represents colony-level fertilization success expressed as a percentage of the maximum achievable fertilization where nearest neighbor distance is zero. Fertilization potential is the proportional area represented by the colored polygon (or equivalently the average of individual colony fertilization success as a percentage of the max).

The fitted model of fertilization success with colony spacing—i.e., the Allee effect (Fig. 2*C* and Table 1)—found that the mean coral population would achieve a maximum theoretical fertilization success, *F_0_*, of ~30%. Applying this value to Eq. **2** gives a mean, population-level fertilization potential of 71% (Fig. 3). To facilitate the use of **2** using our empirical data, we have provided a simpler, cubic fit to our beta-binomial model to aid the calculation of fertilization rate, *F_nn_* and *F_o_* [**1**].

[1] $$F=-0.00222\times {\mathrm{nn}}^{3}+0.1458\times {\mathrm{nn}}^{2}-3.44\times nn+30.$$

## Discussion

This study quantifies the relationship between fertilization success and the isolation of individual corals during spawning of a wild population. We find strong evidence of reproductive Allee effects occurring over a distance of less than 20 m. On average, fertilization success achieved ~30% when colonies were in close proximity (<0.5 m), but this declined rapidly to less than 10% at a separation of 10 m, and virtually zero by 15 to 20 m (though the fitted relationship was slightly higher at a few percent). At the reef scale, average fertilization success varied from 34 to 12% on consecutive nights, which is similar to the few empirical estimates in the literature from plankton tows for eggs postspawning, although less than samples taken from highly concentrated spawn slick aggregations. In the Caribbean, the percentage of fertilized eggs of *Orbicella* spp. peaked at ca. 27% and averaged ca. 8% (14). On the northern Great Barrier Reef, the maximum percentage of fertilized *M. digitata* eggs were, on consecutive nights, 32%, 39%, and 70% (9). In contrast, multispecies spawn slicks on the Great Barrier Reef show 86% fertilization following highly synchronized spawning of high coral cover communities (27). We note that estimates of fertilization success may be higher from spawning slicks than the direct colony-level measurements taken here. First, the eggs of some colonies may not join slicks and be lost, as was observed on both nights. Second, unfertilized eggs can break down in a matter of hours so the apparent fertilization success will increase the later the slick is sampled.

In addition to colony proximity, spawning night had a strong effect on fertilization with a ~threefold decline in success on the second night, despite apparently more extensive spawning activity. Inferring causation is not straightforward because even if we had taken sperm samples they would have been difficult to interpret because of latent sperm from species that spawned earlier, such as *Acropora digitifera*. However, it is feasible, albeit unmeasured, that higher wind speed on the second night led to reduced fertilization. Increased wind intensity enhances vertical mixing of particles through surface shear and turbulence (28). This interaction promotes secondary circulation vortices by combining wind-driven surface currents with wave-induced Stokes drift. As wind speed increases, the size and strength of these vortices also increase, leading to greater mixing within the water column. Such wind-induced vertical mixing of gametes has been described in corals (29) and fish (30). Interestingly, we expected to find that moderately more turbulent conditions might cause greater damage to gametes yet we found higher probabilities of damage on the night with lower wind speed. We hypothesize that the higher proportion of damaged and fragmented gametes might be attributable to the higher rate of fertilization under conditions of lower wind speed and that embryos with 2 to 16 cells are more fragile and more easily broken than unfertilized eggs (EB, GR, CD pers. obs.).

Spawning synchrony influences fertilization success (14) and studies of *Orbicella*, a massive Caribbean species, reveal that clone mates spawn more synchronously than nonclone mates and colonies within 5 m tend to spawn with greater synchrony than those farther apart (31). We also found evidence of small-scale spatial synchrony in the fast-growing table acroporid *A. hyacinthus*. These collective results are intriguing, although the mechanism remains elusive—most likely owing to chemical cues as documented in sea cucumbers (32), or potentially due to extremely localized environmental gradients (33). In principle, local patterns of synchrony reinforce Allee effects by increasing the likelihood of gametes meeting from neighboring colonies. We were unable to test this hypothesis explicitly because we did not attempt to identify the parentage of individual embryos from each maternal colony. We did test the simpler hypothesis that fertilization success is greatest during the middle of a spawning event but that was not supported. Rather, we found that later spawners fared better when conditions were calm, possibly because of a higher local concentration of sperm. Overall, the importance of localized synchrony warrants further study in Pacific corals.

More extensive spawning events, involving a higher proportion of colonies, have resulted in higher gamete concentration and potential for fertilization (9, 14). In these studies, “potential for fertilization” was determined by adding lab-released eggs to water samples obtained from the field. Fertilization rate is proportional to the sample’s sperm concentration, though this does not necessarily imply that eggs would be found in such samples. In addition, plankton tows have been carried out to quantify the proportion of fertilized eggs, particularly after deploying drogues to simulate egg trajectories. Levitan et al. (14) counted the number of spawning colonies observed by divers and found that these were positively associated with both peak fertilization potential and average egg fertilization rate in plankton tows. These results evidence that more extensive events promote higher fertilization success. In our case, we found that any increase in spawning output during night 2 was potentially overwhelmed by the increase in wind. A greater understanding of the contribution of weather to fertilization success is desirable. A potentially deleterious impact of higher wind is consistent with van Woesik’s global analysis that found coral spawning to be associated with predictably calmer periods of wind (34).

The table coral, *A. hyacinthus*, is a highly abundant (0.2 to 0.6 individuals m^−2^) and large-bodied coral capable of extensive gamete release (35). Arguably such species are the least likely type of coral to exhibit reproductive Allee effects. Yet our results suggest precipitous declines in success when colonies become spaced by 8 m and barely any success beyond 15 m. Unfortunately, few data are available on spatial arrangements of this—or any other Pacific coral—let alone that of cryptic morphs or subspecies, with the majority of monitoring programs focusing on overall coral cover, or, at best, distinguishing groups at the genus level. Reporting of coral densities is more common in the Caribbean (*SI Appendix*). Yet, the risk of demographic Allee effects seems feasible, particularly given the high sensitivity of fertilization to coral proximity and the expectation of recurrent bleaching events. Here, we consider the implications of our results for future assessments of coral resilience.

First, we found that density itself is not a good predictor of Allee effects. Unlike nearest neighbor, density does not measure proximity effectively. Any estimate of density is scaled to its sampling unit. Thus, if the sample unit is small—such as a radius of 2 m—then corals in high proximity are included and the metric has some explanatory power. However, this approach ignores any colonies beyond 2 m, which may be close enough to contribute to fertilization. Larger sampling units (e.g., 10 m) are more inclusive but are poorer estimates of proximity unless colonies are arranged regularly, which is rare in nature (36). Field measurements of nearest neighbor are therefore preferred and the recent emergence of photogrammetry as a monitoring tool (37) might facilitate such data collection, though species identification remains challenging. To be clear, we consider the component Allee effect we describe to be density-dependent, but colony density per se might not be the most predictive metric.

Second, site or reef-level estimates of coral population resilience would ideally have some demographic relevance. An example from the fisheries literature is spawning potential ratio, which estimates the lifetime egg production of a fished population and expresses it as a percentage of that same population without fishing (38). For coral, we might replace fishing mortality with that expected from climate impacts and also incorporate the effects of spatial patchiness on local fertilization success (Allee effects). Yet while desirable, such metrics are currently too difficult to create, largely because of difficulties estimating future mortality. However, the spatial patchiness element of such metrics can be approached today, and we provided a simple algorithm to quantify fertilization potential (Eq. **2**). An alternative metric might be the percentage of colonies that meet a desirable threshold of fertilization success, though there remains a challenge in deciding which threshold to pick. A future version of this metric might include colony size, which elevates fertilization success (39).

Given that this is the first estimate of potential Allee effects on a wild coral population, our ability to interpret the metric—at 71%—is limited. Overall, it seems fairly compelling that the spacing among corals at this reef achieves 71% of its theoretical maximum. However, it will be important to examine how this metric varies in different environments, particularly in reefs that have not experienced recent disturbance so that a baseline can be estimated.

While mate limitation is the most frequently invoked mechanism of component-level Allee effects in aquatic invertebrates, the majority of studies—like ours—have evidenced mechanisms without attempting to demonstrate impacts at the population level (5). Yet with sufficient data, it will become clear whether coral fertilization potential falls to a level that results in recruitment failure and negative population growth (i.e., strong Allee effects). In practice, demographic Allee effects are likely to be stronger in some reef environments than others. Isolated reefs with low larval retention are likely to be most at risk because local reproductive failure is less likely to be buffered by external larval supply or the dispersal of larvae from other parts of the same reef that still exhibit high enough population density to generate copious larvae. Thus, further research is needed to establish critical levels of fertilization success, but an opportunity exists for restoration activities to reconnect depleted coral populations and attempt to safeguard their reproductive success.

## Materials and Methods

The study took place during the last 2 wk of March 2024 on the sheltered forereef slope of Uchelbeluu in Palau, Micronesia (Fig. 1*A*). While the area is sheltered from prevailing waves it sits above a steep drop-off that extends to a depth of hundreds of meters. The study site was demarked by following the shallow reef contour (depth ~1 m) for ~100 m and then swimming perpendicularly out toward the drop-off for 60 m. Three days prior to the full moon (24th March 2024), all 210 colonies of *A. hyacinthus* were sampled to determine whether colonies were gravid with eggs and likely to spawn. Two branches were cracked from the middle of each colony to assess egg pigmentation (40). The colonies were scored as immature (i.e., white eggs) which will not spawn during the month, or mature (i.e., pigmented eggs) which are ready to spawn.

All gravid colonies were marked at their base using flagging tape. Although recent studies suggest that *A. hyacinthus* is unlikely to hybridize with other table corals in this location (41), we examined gravidity in *A. cytherea* to be cautious and found white eggs, implying that they would spawn during the following full moon, in April. Moreover, while *A. hyacinthus* does include cryptic species, genetic differentiation has been found to be nonsignificant at the local scale in Palau (42). *A. hyacinthus* rarely creates local clones (<3%), and when it does, these are found within 2 m of one another (43). Had clones occurred at our study site they would be expected to deplete the high fertilization success we observed between colonies in high proximity.

A series of thin nylon strings were established to create five transects that were threaded among gravid colonies and patrolled by individual divers on anticipated spawning nights. That way, five divers could repeatedly check a combined total of 26 colonies each night using red lights (Fig. 1*B*). Separations between colonies and their nearest gravid neighbor ranged from 0.2 to 19 m and all were within one of two depth classes, separated by approximately 2 to 3 m, i.e., a shallow group averaging 2.5 m at high tide and a slightly deeper group averaging 5 m.

**Fig. 4.**
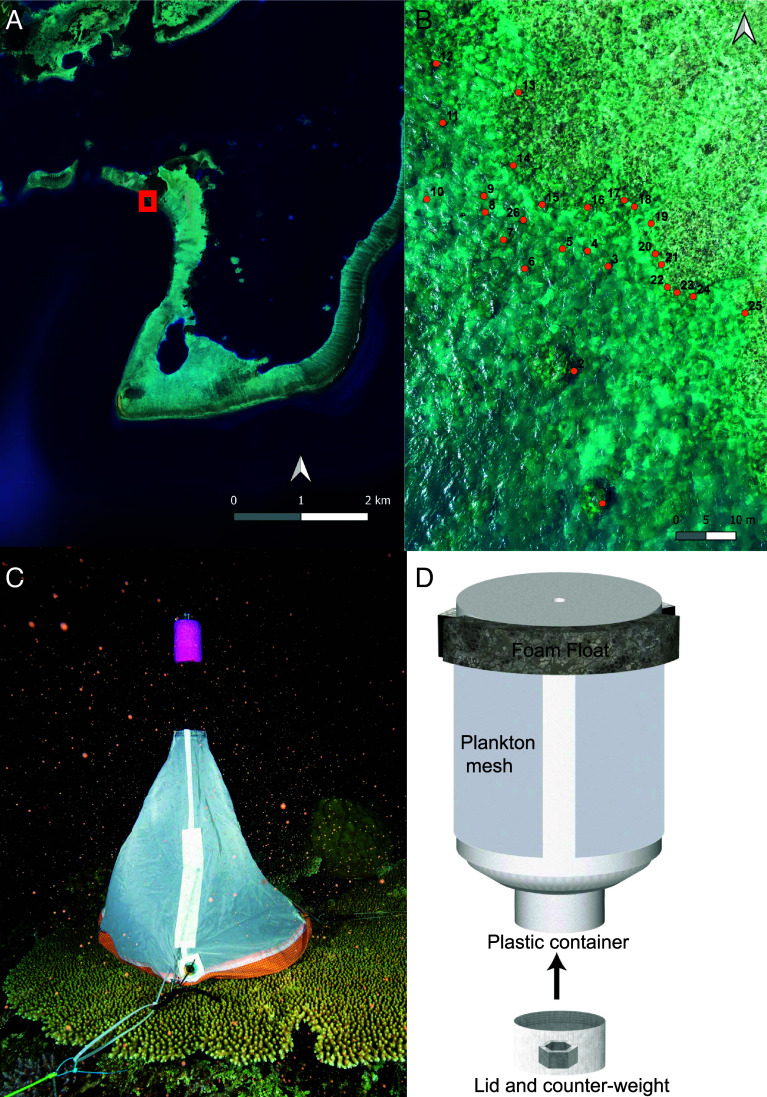
Study design showing Uchelbeluu Reef with the study site marked as a square (*A*), the positions of individual colonies within the study site (*B*), collection hood above *A. hyacinthus* (*C*) just after the egg bundle container (*D*) was released.

At each colony, we created a set of 3 to 4 anchor points for each using resealable cable ties. The anchor points were arranged at approximately 90° positions around the colony and were used to hold spawn catchers, which were placed over colonies within 30 min of anticipated setting—approximately 20:30 local time, based on previous studies at the site (Fig. 4*C*). If no spawning occurred, the spawn catchers were removed the same evening.

Spawn catchers were created using a circular wire frame of basal diameter 0.4 m to which a conical plankton mesh (100 µm) was fitted using eyelets and cable ties and made buoyant with a ring of floatation foam at the apex. The apex formed a plastic tube of ~2 cm diameter into which an egg-sperm bundle container was fixed and held with a hose clamp. The egg-sperm bundle container was created from a 270 mL cylindrical, plastic sample container (height = 95 mm, diameter = 60 mm) to which most of the sides had been removed and replaced by 250 µm plankton mesh (Fig. 1*D*). Our intent was to release a sample of eggs from each colony that could drift at the surface and be fertilized by sperm in situ, thereby allowing us to calculate colony-level fertilization success. The top of the container was sealed and surrounded by a ring of floatation foam to ensure it floated just below the sea surface. Two glow sticks were cable tied to the top.

In the event of spawning, the diver waited until at least 1 cm^3^ of egg-sperm bundles were observed in the top of the container, recording the time that egg-sperm bundle release began. They then released the hose clamp and removed the container, immediately replacing the open-threaded base with a screw cap. The cap held a small lead weight to keep the container vertical. Glow sticks were cracked to fluoresce and the container allowed to rise to the sea surface, whereupon it drifted for at least 60 min prior to collection. Coordinates of each collection point were recorded, and each container placed into a separate holding container that had been filled with seawater prior to any spawning activities (and therefore lacked sperm). On return to the lab, containers were decanted and washed in filtered seawater. Gametes were then fixed by adding 4% buffered formalin in filtered seawater containing 10 g L^−1^ sodium β-glycertophosphate at a ratio of 1:4 fixative to sample. A minimum of 200 eggs were then selected from each container and the proportion of fertilized embryos counted using a dissection microscope. A subset of embryos from colonies that spawned on both nights were also scored to quantify differences in embryo damage, deformity, and fragmentation across the two spawning nights (26th and 27th of March 2024).

In prior spawning events, supplemental experimental fertilization data were collected to provide controls for the experiment. Briefly, two colonies were collected from Uchelbeluu Reef and brought onto a boat upon showing signs of setting. Self-fertilization was assessed by collecting spawned bundles (n = 2-3 replicates) into small containers and assessing fertilization success after ~2 h) and was found to be negligible (0.1%). Although selfing in *Acropora* is possible, it is generally considered low (44) and we consider that it is unlikely to influence trends in our experiment. Further, a single control was conducted to assess that sperm could move freely through the mesh in our containers to fertilize eggs. *Acropora* sperm are generally ~40 µm in flagellum length and ~2 µm width at the head (45) allowing easy passage of the sperm through mesh, and this mesh size is routinely used to separate sperm from eggs in laboratory assays (10, 46). Fertilization success was 72%, which fell within the range of fertilization when the gametes were crossed without a mesh barrier (71 to 92%; n = 6 individual crosses).

Ascent rates of egg containers and individual egg bundles were broadly comparable at 0.03 to 0.06 ms^−1^ vs. 0.06 ms^−1^ respectively (albeit n = 1 per treatment). An earlier experiment that transplanted 20 colonies of *A. hyacinthus* into Palau’s Nikko Bay revealed that the egg containers followed the same trajectory as unconstrained egg bundles released from the same experimental patch. After 70 min, eggs were observed within a meter of the containers. Together with the dye-based study finding comparable dispersal between egg containers and simulated sperm, these observations imply that the experimental containers are a reasonable facsimile of natural gamete dispersal under at least some experimental conditions.

### Effects of Neighborhood Metrics, Spawning Intensity, and Synchrony On Fertilization Rate.

To understand the spatial configuration of *A. hyacinthus* colonies, each gravid colony was identified, planar area calculated, and mapped within a circle of 10 m radius around each of the 26 target colonies used for fertilization measurements. This allowed us to estimate several neighborhood metrics for each of the target colonies. The simplest was nearest gravid neighbor (meters). We also estimated the number of gravid colonies within a 2 m, 5 m, and 10 m radius, respectively (i.e., measurements of local colony density). Finally, we estimated the total area of gravid colonies within a 10 m radius of each target colony. This metric down-weighted colony area exponentially with distance from the target colony in order to reflect the dilution of gametes that would be expected with increased distance from the center.

The adequacy of each neighborhood metric was tested using a beta-binomial model with percentage fertilization success as the response, a continuous neighborhood metric and categorical “night of spawning” as fixed effects, and colony identity (1-26) as a categorical random effect because some colonies spawned on both spawning nights. Model fits were examined using QQ plots and the AIC compared among models. All models were fitted using the package glmmTMB (47).

To compare the intensity of spawning per evening, each of the target colonies was categorized as exhibiting either “no,” “partial,” or “extensive spawning.” Partial spawning has been observed commonly (31) and implied that only a small fraction of the colony released egg bundles; typically less than 20%. We then compared the proportion of “full spawning” occurrences (out of a possible 26) between nights using binomial regression, though several colonies were excluded where the extent of spawning was not reliably observed. To investigate the importance of synchrony in bundle release, we assigned the first observed release on each night as “minute 1” and each subsequent release as a continuous integer corresponding to the relevant minute later (i.e., a value of 10 implies release occurred 9 min later than the first observation). Because we expect synchrony to peak during the center of the event (14), we entered spawning time as a quadratic function nested within night. Depth class (shallow, deeper) was included as an additional effect on fertilization success, following observations that depth can affect synchrony (31).

The effect of spatial distance between colonies on spawning synchrony was tested by regressing the absolute difference in spawn time (minutes) against the pair-wise distance between every pair of colonies that spawned each evening. Small differences in spawn time indicate greater synchrony. To account for the low temporal resolution of most diving computers, which display time to the nearest minute and therefore make synchronization among observers impossible, we included a factor for whether each pair of spawning times were taken by the same observer or different observers.

### Metric of the Allee effect.

The risk of Allee effects can be estimated as the fertilization potential of a local coral population based on its spatial configuration (Eq. **2**). Conceptually, we calculate how closely the coral population could achieve the maximum theoretical fertilization success that would occur when nearest neighbor distances were zero. This is then expressed as a percentage of the maximum. Note that our use of the term “fertilization potential” differs from that in field studies to measure the ability of sperm samples to fertilize eggs (9). The risk of Allee effects at a site is given as

[2] $$\begin{array}{c} \mathrm{Fertilization}\;\mathrm{Potential}\;(\%)=\frac{\sum_{c=1}^{C}\left(\frac{F_{{\mathrm{nn}}_{c}}}{F_{0}}\times 100\right)}{C}, \end{array}$$

where percentage fertilization rate *F* is entered for every colony *c*, from its nearest neighbor, *nn*, and expressed relative to the maximum fertilization rate at a separation of zero (*F_0_*). The metric is summed across all *C* colonies and divided by *C* to yield a mean. Essentially, this is the average colony fertilization rate as a function of the maximum possible fertilization rate.

## Supplementary Material

Appendix 01 (PDF)

## Data Availability

Rscripts and data have been deposited in Github (https://github.com/MSEL-UQ/Allee-effects-Mumby-et-al.git) (48).
