# Supplementary material for: Allee effects limit coral fertilization success
Source: Proc Natl Acad Sci U S A. 2024 Dec 16;121(52):e2418314121. doi: 10.1073/pnas.2418314121 (PMC11670066; doi:10.1073/pnas.2418314121)
Supplement: Supplementary file 1 — Appendix 01 (PDF) [file pnas.2418314121.sapp.pdf]

PNAS

Supporting Information for

Allee Effects Limit Coral Fertilization Success

Peter J Mumby[1,2*], Greta Sartori[2], Elizabeth Buccheri[1, 3], Cinzia Alessi[2], Hannah Allan[1, 3], Christopher Doropoulos[3], Geraldine Rengiil[2], Gerard Ricardo[1, 3]

*Correspondence: Peter Mumby
Email: p.j.mumby@uq.edu.au

This PDF file includes:

Supporting text
Tables S1 to S2
SI References

Other supporting materials for this manuscript include the following:

Link to Datasets

Supporting Information Text

1) Self-fertilization tests

Test of self-fertilization in *Acropora hyacinthus* (Table S1). Twenty colonies were removed from the study site in Palau in March 2023. These were transferred to Nikko Bay for an experiment and were the only corals spawning in the bay on the nights observed.

Two colonies were removed to the boat an hour before spawning and 2-3 samples of egg/sperm bundles placed in separate containers once each colony spawned. Each sample was fixed approximately 2 hours later and eggs examined for evidence of self-fertilization. Four of the samples had zero fertilized embryos (of 156-173 eggs per sample) and one had a single fertilized egg within 184 sampled eggs (i.e., fertilization rate of 0.005). We conclude that self-fertilization, while not impossible, is rare and is unlikely to have impacted our experimental results.

Table S1. Self-fertilization in *Acropora hyacinthus* in Palau 2023

Date	Colony	Sample	Number of eggs	Number of embryos	Total	Fertilization rate (proportion)
22/03/2023	A	1	156	0	156	0
22/03/2023	A	2	156	0	156	0
22/03/2023	A	3	163	0	163	0
22/03/2023	B	1	183	1	184	0.005
22/03/2023	B	2	173	0	173	0

2) List of studies reporting coral species colony densities (Table S2)

Table S2. Summary of studies report the density of coral colonies by species

**Species**	Growth form (e.g. digitate)	Survey year	Location	**Colonies/m²**	Mean nearest neighbour/ intercolonial	Coral cover (%)	Clustering (uniform, random, clustered)	Size (m^2)	Juveniles included?	**Reference**	Notes
**Acroporidae**											
Acropora palmata	Branching/ encrusting			0.07 to 0.28	NA		NA			(1)	
Acropora cervicornis	Branching			<1.22		<2				(2)	Symposia proceedings
Acropora palmata	Branching			<1.25		25				(2)	Symposia proceedings
Acropora yongei	Arborescent	2015-2016	Southern Ryukyu Archipelago, Japan	0.0006 –0.029	3.6 ± 0.3 –20.7 ± 5.2					(3)	
Acropora hyacinthus	Table	2014	Moorea	0.073±0.023						(4)	10 m depth only
**Pocilloporidae**											
Madracis mirabilis	Branching			9.9 ± 2.2		1.2 ± 0.27				(5)	
Pocillopora spp.				21.81 ± 1.56					Juv. Density 2.56 ± 0.89	(6)	Genus level – no species defined
Seriatopora hystrix	Branching			0.5						(7)	
Merulinidae											
Montastrea annularis				20						(8)	Documented declines over time

Orbicella annularis	Massive			8 to 51						(9)	*Note mostly small size class
Agariciidae											
Agaricia agaricites	Foliose			2.1 ± 0.4		0.5 ± 0.09				(5)	
Poritidae											
Porites astreoides	Massive	1999		1.7 ± 0.2					Yes	(10)	
Mussidae											
Diploria strigosa		2003		0.35 ± 0.04						(10)	
Diploria strigosa		2007		0.98 ± 0.17						(10)	

SI References

1. Williams D, Miller M, & Kramer K (2008) Recruitment failure in Florida Keys Acropora palmata, a threatened Caribbean coral. *Coral Reefs* 27:697-705.
2. Miller S, Chiappone M, Rutten LM, & Swanson DW (2008) Population status of Acropora corals in the Florida Keys.
3. Zayasu Y & Suzuki G (2019) Comparisons of population density and genetic diversity in artificial and wild populations of an arborescent coral, Acropora yongei: implications for the efficacy of "artificial spawning hotspots". *Restor. Ecol.* 27(2):440-446.
4. Ladd MC, Winslow EM, Burkepile DE, & Lenihan HS (2021) Corallivory varies with water depth to influence the growth of Acropora hyacinthus, a reef-forming coral. *Ecosphere* 12(8):e03623.
5. Brito-Millán M, Vermeij MJ, Alcantar EA, & Sandin SA (2019) Coral reef assessments based on cover alone mask active dynamics of coral communities. *Mar. Ecol. Prog. Ser.* 630:55-68.
6. Edmunds PJ, Nelson HR, & Bramanti L (2018) Density-dependence mediates coral assemblage structure. *Ecology* 99(11):2605-2613.
7. Warner PA, Willis BL, & Van Oppen MJ (2016) Sperm dispersal distances estimated by parentage analysis in a brooding scleractinian coral. *Mol. Ecol.* 25(6):1398-1415.
8. Edmunds PJ & Elahi R (2007) The demographics of a 15-year decline in cover of the Caribbean reef coral Montastraea annularis. *Ecol. Monogr.* 77(1):3-18.
9. Edmunds PJ (2019) The demography of hurricane effects on two coral populations differing in dynamics. *Ecosphere* 10(9).
10. Edmunds PJ (2010) Population biology of Porites astreoides and Diploria strigosa on a shallow Caribbean reef. *Mar. Ecol. Prog. Ser.* 418:87-104.

3) Link to Datasets:

Underlying data and R scripts for this paper can be accessed at:

https://github.com/MSEL-UQ/Allee-effects-Mumby-et-al.git
